# Transient elastography and APRI score: looking at false positives and false negatives. Diagnostic performance and association to fibrosis staging in chronic hepatitis C

**DOI:** 10.1590/1414-431X20165432

**Published:** 2016-08-01

**Authors:** L.C. Mendes, P.A. Ferreira, N. Miotto, L. Zanaga, E. Gonçales, M.S. Lazarini, F.L. Gonçales, R.S.B. Stucchi, A.G. Vigani

**Affiliations:** 1 Departamento de Doenças Infecciosas, Universidade Estadual de Campinas, Campinas, SP, Brasil; 2 Departamento de Doenças Infecciosas, Universidade Federal de São Paulo, São Paulo, SP, Brasil

**Keywords:** Liver biopsy, Noninvasive tests, APRI, Transient elastography, Accuracy, False results

## Abstract

Although long regarded as the gold standard for liver fibrosis staging in chronic hepatitis C (CHC), liver biopsy (LB) implies both the risk of an invasive procedure and significant variability. The aim of this study was to evaluate the diagnostic performance for transient elastography (TE) and aspartate aminotransferase to platelet index (APRI) used alone and in combination compared to liver biopsy and to analyze false positive/negative results. Patients with CHC, and no previous clinical diagnosis of cirrhosis were enrolled to undergo liver biopsy, TE and APRI. A total of 182 adult patients with a median age of 55 years and median body mass index of 26.71 kg/m^2^ were analyzed. On LB, 56% of patients had significant levels of fibrosis (METAVIR F≥2) and 28% had advanced fibrosis (F3/F4). The strongest performance for both tests was observed for exclusion of advanced fibrosis with good negative predictive values (89 and 86%, respectively). Low necroinflammatory activity on LB was associated with false negative TE. False positives were associated with NASH and smaller LB fragments. Correlation between APRI and Fibroscan for F≥2 was 100% and 84% for F≥3 and remained high in both false negative and false positive instances, correctly identifying F<2 in 71% of cases and F<3 in 78% (and potentially foregoing up to 84% of LB). We concluded that low individual performance indicators could be attributable to limitations of LB. Poorer differentiation of lower levels of fibrosis is a known issue for LB and remains so for noninvasive tests. Good predictability is possible, however, for advanced fibrosis.

## Introduction

Liver fibrosis (LF) staging is an important component of chronic hepatitis C (CHC) management. While patients exhibiting minimal or absent fibrosis progress slowly over a long period of time, those with advanced fibrosis (septal bridging or regenerative nodules) will almost invariably progress to clinical cirrhosis in less than 10 years. Furthermore, LF is a major prognostic factor in CHC, directly correlating to the risk of developing liver-related complications and death (1,2).

Although long regarded as the gold standard for fibrosis staging, liver biopsy (LB) has limitations both in diagnostic performance (either because of sampling error or observer variability) (3) and regarding safety concerns, with 0.3 to 0.6% overall risk for complications and a 0.05% mortality rate (4). Notwithstanding, biopsy holds to this day a paramount role in the diagnosis and management of liver disease, as it can offer invaluable information regarding inflammatory activity, steatosis, steatohepatitis, and coexisting morbid conditions such as iron overload, auto immune hepatitis features, among others.

In an attempt to overcome potential risks and expand access and eligibility in LF staging, several noninvasive approaches have been developed (5–8), some relying on analysis of physical changes associated with liver fibrosis, such as elastography, and others on biochemical markers and scoring systems ranging from isolated platelet counts (9) to more elaborate indexes, such as Fibrotest^®^, Fibrometer^®^, and Hepascore^®^. These indexes have variable diagnostic performances (10–12), usually with stronger predictability for advanced fibrosis and cirrhosis when compared to significant fibrosis or specific METAVIR level staging.

Aspartate aminotransferase (AST) to platelet ratio index (APRI) is one of the most validated and simple-to-use scoring systems for fibrosis prediction and has been reported to achieve areas under the receiver operating curves (AUROCs) for the diagnosis of significant fibrosis, advanced fibrosis and cirrhosis of 0.77, 0.80 and 0.83, respectively (13). However, those levels of predictability are conditioned to optimal thresholds that occur in less than 30% of patients (14).

Imaging techniques have also been developed such as elastography, which measures liver stiffness (LS) and correlates with liver fibrosis. Transient hepatic elastography (TE) (Fibroscan^®^, Echosense, France) uses mechanic shear wave velocity measurements through monodimensional ultrasound (15), with 0.79, 0.91 and 0.97 AUROCs for significant fibrosis, advanced fibrosis and cirrhosis, respectively (16). However, previous reports have established limited resolution in lower levels of fibrosis and in patients with larger abdominal circumferences. Other limitations of Fibroscan include ascites and physiological or pathological processes associated with liver congestion. Also, cost considerations still make elastography inaccessible to many resource-limited areas.

Several attempts have been made to improve diagnostic performances and likelihood ratios by combining different tests and possibly overcoming their individual limitations. Associating test modalities in a sequential or synchronous approach can provide up to 85–90% predictability for significant fibrosis or advanced fibrosis/cirrhosis (17–19).

Both elastography and biochemical scores present promising noninvasive approaches for complementing or even substituting histological analysis. Overall diagnostic performance across different patient populations, however, remains questionable. Moreover, correlation between TE and APRI, and potential uses for combined diagnosis in clinical practice remain unclear. The aim of this study was to evaluate diagnostic performances of APRI and TE, alone and in combination, in a Brazilian CHC population to detect significant fibrosis (F≥2) or advanced fibrosis (F≥3) and to determine if LB could potentially be avoided in a proportion of cases.

## Material and Methods

### Patient enrollment and data collection

For this prospective cross-sectional study, adult patients (>18 years) with CHC, followed in an outpatient university hospital clinical setting (Ambulatório de Hepatites Virais of the Universidade Estadual de Campinas) from January 2013 to June 2015, were included. CHC was defined as positive detection of hepatitis C virus (HCV) RNA (Abbot Real Time HCV Abbott Laboratories, Germany) in serum samples obtained at least 6 months after initial seropositivity for antibodies against HCV.

Exclusion criteria were co-infection with human immunodeficiency virus (HIV) or hepatitis B virus (HBV), decompensated liver disease (presence or history of ascites), hepatic encephalopathy, portal hypertension-related bleeding or hepatocellular carcinoma (HCC), prior liver transplantation or patients with clinical, radiological or endoscopic diagnosis of cirrhosis (such as direct or indirect evidence of portal hypertension).

For all patients, anthropomorphic data were collected comprising gender, weight, height, body mass index (BMI), and waist and thoracic circumferences. Serum samples were obtained on the same day of liver biopsy and subjected to routine laboratory biochemical techniques for dosing of AST, alanine aminotransferase (ALT), and platelet counts.

### Histological evaluation

LB was performed percutaneously after local anesthesia and mild sedation with a 14 gauge tru-cut needle. Liver specimen fragments were considered acceptable at a minimum of 15 and preferable 25 mm length. Histological analysis was performed by a blinded senior institutional liver pathologist after formalin fixation, paraffin embedment, hematoxylin-eosin and Masson's-Trichrome staining for all samples and scored according to the METAVIR fibrosis staging system (20). Fibrosis was either absent (F0); confined to portal spaces without septa (F1); extending beyond portal spaces with few portal-portal, portal-center, center-center septa (F2); extending beyond portal spaces with numerous septa (F3) or diffuse with numerous septa and formation of regenerative nodules - cirrhosis (F4). Significant fibrosis was defined as F≥2 and advanced fibrosis as F≥3.

### Noninvasive tests

TE measures were obtained with Fibroscan^®^, model 502 (Echosense) M probe, after 2-h fasting, on the right lobe, through intercostal spaces with the patient in a supine position. The unique blinded operator for TE measurements was experienced in more than 100 prior examinations, as recommended (16). LS values were included in the analysis with at least 10 valid measures, over 70% success rate and interquartile range (IQR) less than 30% of the median value of LS measures. Significant fibrosis was defined as LS above 7.1 kPa and advanced fibrosis as LS above 9.5 kPa. Cirrhosis was diagnosed when LS results were over 12.5 kPa. Discordance with LB was defined as non-agreement on the basis of defined parameters (significant fibrosis and advanced fibrosis).

APRI was calculated from components obtained on the day of liver biopsy using the formula described in the literature (AST in IU·mL^-1^·upper limit of normality^-1^)/platelet count (10^9^/L). Significant fibrosis was defined as highly unlikely when APRI was less than 0.5 and as highly likely if APRI result was higher than 1.5. Advanced fibrosis/cirrhosis was considered as highly improbable if APRI was lower than 1.0 and as highly probable if APRI was higher than 2.0 (13). Noninvasive test performance indicators were calculated using histological analysis as the gold standard method.

### Statistical analysis

The study population was analyzed with descriptive statistical analysis using Epi-info version 3.5.4 (CDC, USA) and OpenEpi version 3.03a (Emory, USA). Continuous variables were analyzed with Student's *t*-test or Mann-Whitney test, where appropriate. Categorical variables were compared using the chi-square test. Diagnostic performances for different tests were analyzed separately and in combination according to sensitivity (Se), specificity (Sp), negative predictive value (NPV) and positive predictive values (PPV), positive likelihood ratio (LR+), negative likelihood ratio (LR−), accuracy (Ac), Cohen's Kappa correlation value (κ) and AUROC. Test combinations were evaluated following a sequential approach to potentially establish absence or presence of significant (F≥2) or advanced (F≥3) fibrosis. Finally, using an 85% accuracy threshold for predictability, the number of liver biopsies potentially avoided were calculated. Baseline continuous data are reported as medians, and categorical variables are reported as frequencies or percentages. Univariate analyses were performed using chi-square, Fisher, and analysis of variation or Mann-Whitney, as appropriate. P<0.05 was considered to be statistically significant.

### Ethical considerations

Study design, protocols, patient enrolment, and data collection and storage were in accordance with ethical considerations supported by the updated 1975 Declaration of Helsinki. Patients were included in the study after written informed consent was obtained. The study was reviewed and approved by the Ethics Committee for Research of the School of Medical Sciences, State University of Campinas (UNICAMP).

## Results

During the study period, 198 patients were eligible according to inclusion criteria. Of those, 12 (6,1%) were excluded due to liver biopsy fragments with less than 15 mm and 4 (2,0%) were excluded after LS measurements were considered invalid according with the described criteria. A total of 182 patients were included in the final analysis.

### Demographic and clinical characteristics

Patient characteristics are presented in Table 1. Median age was 55 years, 82 (70%) were Caucasians and 73 (61%) were male. According to abdominal circumferences, 16 (5.5%) patients were considered to be obese; according to BMI, 18 (9.9%) patients were characterized as obese.

**Table:**
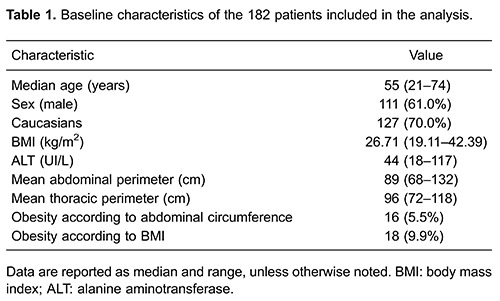

### Histological analysis

LB procedures did not result in any serious adverse outcome. LB fragments median length was 18 mm and was greater than 25 mm in 28% of samples. Mean number of portal tracts was 9.8 (from 6 to 13). Histological analysis results according to METAVIR scoring system revealed that 15 (8.0%) patients were F0, 63 (35.0%) were F1, 50 (28.0%) were F2, 45 (25.0%) were F3, and 9 (5.0%) were F4. Mild steatohepatitis was found in 11 (6.0%) patients, moderate steatohepatitis in 6 (3.3%) and severe steatohepatitis in 4 (2.2%). Necroinflammatory activity was absent (A0) in 21 patients (11.0%), mild (A1) in 69 (38.0%), moderate (A2) in 73 (41%) and severe in 19 (10.0%).

### Serum markers scoring system

**Figure 1:**
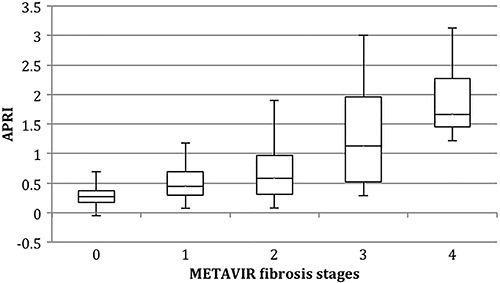
Box plot distribution of aspartate aminotransferase to platelet index (APRI) results according to METAVIR LB staging. First and third quartiles are represented as top and bottom of the boxes, and the error bars show minimum and maximal values. The vertical length of the box represents the interquartile range and the horizontal line through the middle represent the median value.

APRI results were classifiable in 57% of cases, which were deemed as highly unlikely for significant fibrosis in 87 (48%) of patients and as highly likely in 16 (9%). Fibrosis level distribution among different APRI results are shown in Figure 1. Specificity was 95% with LR+ of 5.18, and AUROC of 0.71; other diagnostic performance indicators are shown in Table 2.

**Table:**
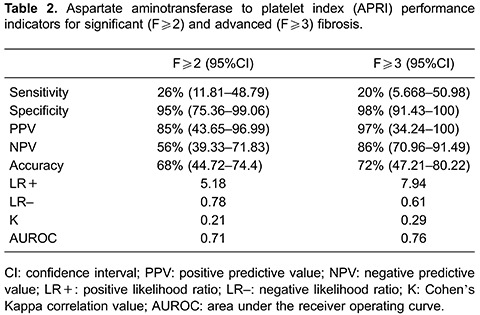

**Table:**
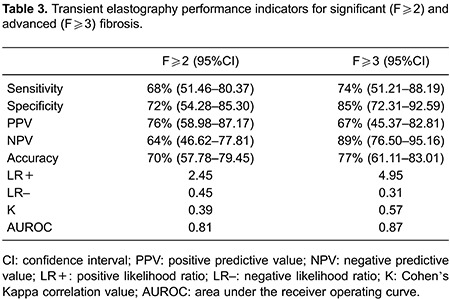

APRI classification for advanced fibrosis/cirrhosis was possible in 77% of patients (APRI <1.0 or >2.0). F≥3 was found to be likely (APRI>2.0) in 7 patients (4%) and unlikely (APRI<1.0) in 134 (74%) patients, with 98% specificity and 97% PPV with AUROC of 0.76.

### Transient elastography

**Figure 2:**
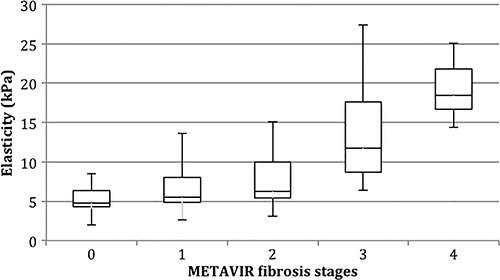
Box plot distribution of Fibroscan results according to METAVIR LB staging. First and third quartiles are represented as top and bottom of the boxes, and the error bars show minimum and maximal values. The vertical length of the box represents the interquartile range and the horizontal line through the middle represent the median value.

Mean time interval from LB to TE was 4.2 months (range 0.5 to 7 months). In terms of quality control, IQR/median presented high homogeneity with a median value of 10% (range 7 to 16%) and 97% mean success rate. LS values ranged from 2.6 to 42.8 kPa (median, 7.1 kPa). TE showed significant fibrosis in 87 (48%) patients and advanced fibrosis/cirrhosis in 57 (30%), as displayed in Figure 2. For significant fibrosis, sensitivity was 68% with PPV of 76% and NPV of 64%, and AUROC of 0.81. For advanced fibrosis/cirrhosis specificity was 85% with NPV of 89%, AUROC 0.87 and high correlation (κ =0.57), as shown in Table 3.

### Combination of diagnostic tests

**Figure 3:**
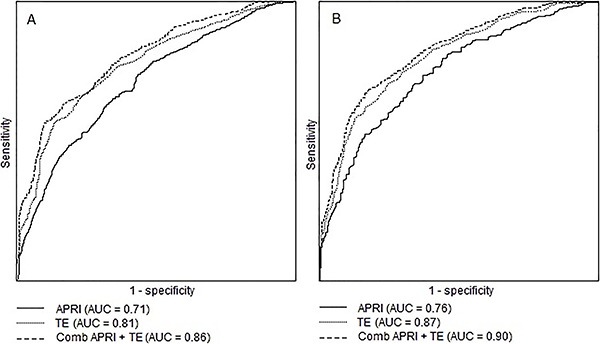
Receiver-operating-characteristic (ROC) curves of noninvasive tests transient elastography (TE), aspartate aminotransferase to platelet index (APRI) and a combination of both tests for significant (*A*) and advanced (*B*) fibrosis in chronic hepatitis C patients compared to histological analysis through liver biopsy.

Correlation between APRI and TE for F<2 was 100% and, for F<3, 98.4%. Combining both tests successfully identified patients without significant fibrosis in 78% of times (κ=0.34, AUROC 0.86), and patients without advanced liver fibrosis in 84% of cases (κ=0.38, AUROC 0.90). AUROCs for individual tests as well as for combinations are shown in Figure 3.

In order to stage patients using significant fibrosis as a diagnostic target (F≥2), combining APRI and TE could have avoided LB in 54.5% of cases. For advanced fibrosis/cirrhosis prediction, combination of TE and APRI would have bypassed 76.3% of LB.

### Factors associated with test performances

Overall accuracy (AUROCs) was not significantly affected by LB fragment length (>20 *vs* <20 mm, P=0.31), median ALT levels (>50 *vs* <50 UI/mL, P=0.2) or necroinflammatory activity grading in histological analysis (P=0.19). Low BMI (P=0.022) and smaller waist circumferences (P=0.031) were associated with greater AUROCs for TE (Table 4).

**Table:**
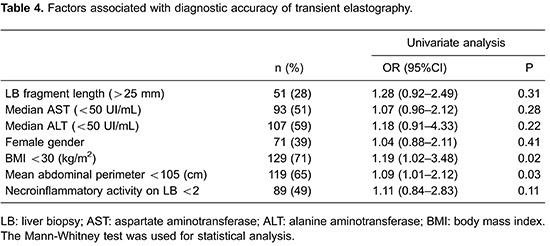

### False negative TE results

For significant fibrosis, false negative TE results comprised 32% of the study sample. On histological analysis, 83% were classified as F2 and 7% were found to be F4. Of note, 52% received non-classifiable APRI results (>0.5 and <1.5), 48% were also predicted to be F<2 on APRI and none had APRI>1.5. On univariate analysis, lower necroinflammatory activity (A<2 *vs* A≥2, according to METAVIR system) was associated with false negative TE results (OR=2.03, 95%CI=1.17–3.69). For advanced fibrosis, 26% of patients falsely tested negative on TE, 82% of which were F3 on LB. Of these, 79% had concordant APRI results and the remainders were non-classifiable (none had APRI<1.0).

### False positive TE results

Among 28% of patients who tested positive on TE for significant fibrosis and were found to be F<2 on LB, 80% were F1 and 83% tested positive as well on APRI. Liver fragments of less than 20 mm were associated with false positive results on TE for significant fibrosis (OR=2.58, 95%CI=1.79–7.22). For advanced fibrosis, of the 15% false positives 78% were classified as F2 on LB and 22% as F1. APRI results also were positive for advanced fibrosis on 93% of these patients and, on multivariate analysis, NASH diagnosed in LB was a moderate predictor of false positive TE results (OR=1.98, 95%CI=1.17- 4.11).

### False negative APRI results

Fifty-four percent of patients that tested negative on APRI for significant fibrosis were classified as F≥2 on LB, 68% of which were F2 and 5% were F4. TE results were also negative for significant fibrosis in 83% of cases. Considering advanced fibrosis, 60% of negative APRI results were found to be false negatives, 77% of which were classified as F3 on LB. Of these, 94% also had negative TE results. Age of less than 50 years was associated with false negative APRI results for significant fibrosis (OR=1.78, 95%CI=1.02–3.61).

### False positive APRI results

Overall there were few instances of false positive APRI results. For significant fibrosis, 5% of patients falsely tested positive on APRI, all of whom were found to be F1 on LB but, on the other hand, also tested positive on TE. Only 2% of patients tested positive on APRI for advanced fibrosis and had LB results of F<3 (66% were F2 and 33% were F1), all of whom tested positive on TE.

## Discussion

We present a prospective cross-sectional study aimed to evaluate the performance of two noninvasive LF staging tests individually and combined, in an outpatient CHC population in Brazil comprising 30% of patients with advanced fibrosis. False positive and false negative results and their correlated characteristics were also evaluated.

Among different technologies for noninvasive LF staging, LS determination using TE has been extensively investigated in recent years (10,16,21) and therefore was elected as one of the objects of our study. In the CHC population, diagnostic performance indicators showed good performance for ruling in and ruling out significant or advanced fibrosis and cirrhosis. For significant fibrosis (F≥2), AUROC ranged from 0.85 to 0.91; for advanced fibrosis (F≥3), AUROC ranged from 0.87 to 0.92 and, for cirrhosis, from 0.87 to 0.95. Our results are in concordance with previous findings, showing moderate NPV for F≥2 (64%) and good NPV for F≥3 (89%) with 0.81 and 0.87 AUROCs for significant and advanced fibrosis, respectively. Differentiating specific levels of fibrosis according to METAVIR scoring system, however, was shown to be challenging for TE (22,23), especially in the lower levels (F0 *vs* F1 and, to a lesser degree F1 *vs* F2). Advanced fibrosis and cirrhosis prediction is well accomplished by TE, with best performances in ruling out F≥3 (24). Detection of significant fibrosis is somewhat poorer than for cirrhosis or advanced fibrosis (AUROC 0.84 *vs* 0.94, respectively) (25). Diagnostic performance for TE was significantly influenced by BMI<30kg/m^2^ and lower abdominal perimeters. Study design precluded the use of XL probes due to low reproducibility in previous studies (26,27). M probe performance in obese and overweight patients is indeed inferior, with more inconclusive and invalid test results.

APRI was the second diagnostic approach we studied because it is a simple biomarker index and the most widely available for predicting LF (13). It has been extensively evaluated in CHC for diagnosis of significant fibrosis and cirrhosis with different cut-off values (28). For exclusion of significant fibrosis, results of less than 0.4 carry the greater sensitivity (88%), however, the 0.5 cut-off for F<2 is the most well studied (23 studies and 4,595 patients) with 74% sensitivity and 49% specificity. APRI >1.5 is the optimal cut-off level for diagnosing significant fibrosis (95% specificity). For advanced fibrosis, APRI <1.0 carries an 81% NPV (AUROC 0.80) and, for APRI >2.0, the specificity for F≥3 is 93%. Comparing APRI and LB for significant fibrosis and advanced fibrosis/cirrhosis our results present slightly lower diagnostic power (AUROC 0.78 and 0.82, respectively), although APRI had very good correlation with TE results for both F<2 and F<3 (83 and 94% of cases, respectively).

LB was used as the gold-standard test against which others were compared. In recent years, attention has been drawn to the fact that histological assessment of liver specimens has its pitfalls and disadvantages. Sampling error may be an issue considering the limited hepatic tissue extension represented in a sample (1/50,000 of actual mass) ranging from 45 to 55% according to fragment length (3). Furthermore, as a highly operator-dependent test, inter-observer variability has been shown to reach 35% (3,29,30), with differences among reports of up to 2 degrees of fibrosis. Intra-pathologist variability has also been reported to be as high as 30%. Variability also occurs when examining right and left lobes of the liver separately with up to 33% discordance. Between two fragments of at least 15 mm taken from the same puncture site there was discordance of 1 or more fibrosis stages in 45% of cases (3,30,31).

Analyzing false negative TE results, significant fibrosis prediction was moderately impaired by lower levels of necroinflammatory activity on LB examination. Regarding APRI, conversely, false negative results for significant fibrosis were moderately influenced by age (less than 50 years). On the other hand, false positive on TE for significant fibrosis was influenced by comparison to LB results from fragments of less than 20 mm, which, as previously described (6) carries the highest probability of under staging in histological analysis. Also, presence of NASH on LB was an independent predictor of false positive TE for significant fibrosis, possibly acting as a confounder both on LS parameters and associated inflammatory activity. Most importantly, both false negative and false positive results of APRI and TE were highly correlated, especially in lower levels of fibrosis, signaling a possible important role of LB limitations as a determinant of the somewhat low performance indicators of noninvasive tests.

Combining results of APRI and TE has not been extensively explored in past studies. We found that concordance between APRI and TE was 100 and 98.4% for significant fibrosis and advanced fibrosis, respectively, in spite of somewhat low individual diagnostic performance indicators. In agreement with other authors (20,32), limitations of histological analysis and possible sampling errors intrinsic to LB were considered in our study to be the main contributing factor for noninvasive tests accuracy results. Furthermore, the association of TE and APRI provides a reasonably cost-effective approach to LF staging in resource-limited settings in comparison to previously reported algorithms that combine LS with patented scores such as Fibrotest.

In terms of study design, examination by a single liver pathologist blind to the results of comparator tests represents a weakness in our study. Previous reports demonstrate that experienced liver pathologists can produce LB examinations with over 20% of serious misclassifications (2 degrees of fibrosis according to the METAVIR system) (33). For that reason, many different groups (1012,) employed double blind reading by different independent pathologists, usually resorting to a third one for discordant results. Analytical limitations notwithstanding, single pathologist examination provides a more accurate depiction of real life in clinical settings and decision-making scenarios.

Another recognizable limitation of our population sample is the low incidence of cirrhosis identified through histological analysis. That in itself could lower accuracy values for comparator tests. However, we have chosen to elect the combined surrogate marker of advanced fibrosis, comprising both METAVIR F3 and F4 stages, which has been previously shown to correlate well with disease progression among other clinical outcomes (34,35).

As previously observed, our results point to stronger diagnostic performances in ruling out significant or advanced fibrosis in the CHC population for the two studied tests used individually or in combination. LB avoidance needs not necessarily to be the only desired outcome for noninvasive tests incorporation in clinical practice. In fact, the use of noninvasive tests can also play a pivotal role in providing pre-test probabilities for critical and empowered interpretation of histological analysis results in different clinical scenarios. Undeniably, LB provides invaluable information for clinical decision-making, such as necroinflammatory activity and steatohepatitis, which was present in 11% of our study population and could otherwise have remained undiagnosed. Future research opportunities for further understanding performance indicators for noninvasive tests or algorithms should consider correlation between different modalities and the imperfect gold standard against which they are challenged.

Nonetheless, specific METAVIR level staging remains challenging across all noninvasive test modalities, with very low accuracy and poor correlation among different markers. Also, for most serum markers and for TE, diagnostic performance is stronger in advanced levels of fibrosis than for discriminating significant *vs* non-significant fibrosis. However, considering the currently shifting paradigm of CHC treatment from the ages of interferon-based therapies with low success rate and unfavorable safety profiles to highly efficacious and largely well tolerated directly acting antivirals, diagnosing specific levels of fibrosis tends to be rendered less important in supporting decision-making in clinical practice. In fact, deciding on antiviral treatment indication will perhaps rely less on determining patients who can await longer than others, but rather on tailoring specific therapeutic regimens for advanced levels of fibrosis or cirrhosis, as well as initiating recommended screening procedures, all of which are suitable for noninvasive tests either isolated or in combination.

Our results go beyond establishing diagnostic performance through conventional indicators. We have analyzed discordant results between noninvasive tests and LB and determined associated predictive factors, as well as documented a strong correlation between serum biomarkers and TE. Considering LB sample- and operator-related variability, we suggest that accuracy-based performance standards for noninvasive methods may be, in fact, conditioned by an imperfect gold standard. Taking into account possible interfering factors such as necroinflammatory activity or NASH, TE and APRI, and especially their combined results can potentially improve diagnostic capabilities.
